# Molecular profiling of stem cell-like female germ line cells in *Drosophila* delineates networks important for stemness and differentiation

**DOI:** 10.1242/bio.046789

**Published:** 2019-11-12

**Authors:** Manu D. Tiwari, Daniela M. Zeitler, Gunter Meister, Andreas Wodarz

**Affiliations:** 1 Molecular Cell Biology, Institute I for Anatomy, University of Cologne Medical School, Kerpener Str. 62, 50937 Köln, Germany; 2 Cluster of Excellence – Cellular stress response in aging-associated diseases (CECAD), University of Cologne, Joseph-Stelzmann-Str. 26, 50931 Cologne, Germany; 3 Stem Cell Biology, Institute for Anatomy and Cell Biology, Georg-August University Göttingen, Justus-von-Liebig-Weg 11, 37077 Göttingen, Germany; 4 Regensburg Center for Biochemistry (RCB), University of Regensburg, Universitätsstr. 31, 93053 Regensburg, Germany; 5 Center for Molecular Medicine Cologne, University of Cologne, Robert-Koch-Str. 21, 50931 Cologne, Germany

**Keywords:** Stem cells, RNA-seq, *Drosophila*, RIP-seq, Transcriptome

## Abstract

Stem cells can self-renew and produce daughter cells destined for differentiation. The precise control of the balance between these two outcomes is essential to ensure tissue homeostasis and to prevent uncontrolled proliferation resulting in tumor formation. As self-renewal and differentiation are likely to be controlled by different gene expression programs, unraveling the underlying gene regulatory networks is crucial for understanding the molecular logic of this system. In this study, we have characterized by next generation RNA sequencing (RNA-seq) the transcriptome of germline stem cell (GSC)-like cells isolated from *bag of marbles* (*bam*) mutant *Drosophila* ovaries and compared it to the transcriptome of germ line cells isolated from wild-type ovaries. We have complemented this dataset by utilizing an RNA-immunoprecipitation strategy to identify transcripts bound to the master differentiation factor Bam. Protein complex enrichment analysis on these combined datasets allows us to delineate known and novel networks essential for GSC maintenance and differentiation. Further comparative transcriptomics illustrates similarities between GSCs and primordial germ cells and provides a molecular footprint of the stem cell state. Our study represents a useful resource for functional studies on stem cell maintenance and differentiation.

## Abstract (precis)

**Summary:** Fruit fly germline stem cell differentiation is accompanied by major changes of the transcriptome that may be regulated at the post-transcriptional level.

## INTRODUCTION

Since being first isolated and *in vitro* propagated from mouse blastocysts in 1981, stem cells have piqued considerable scientific interest and captivated the society, albeit with a fair share of debate (Baylis and McLeod, 2007; Evans and Kaufman, 1981; McLaren, 2001; Watt and Driskell, 2010). Stem cells are undifferentiated, mitotically active cells that can divide either stochastically or deterministically to renew themselves and produce progeny with restricted developmental potential (Morrison et al., 1997). Their hallmark self-renewal is essential for tissue maintenance in multicellular organisms and has for a long time held considerable promise for regenerative cell therapies (Singec et al., 2007).

All this enthusiasm for stem cells has been propelled by advances in stem cell biology, which have been fueled and complemented by research on model organisms (Hunter, 2008). For instance, the existence of the so-called stem cell niche as a microenvironment essential for stem cell sustenance was first discovered in ovaries of *Drosophila melanogaster*, and has since been observed in many different tissues across the plant and animal kingdoms (Morrison and Spradling, 2008; Xie and Spradling, 2000). Each of a pair of *Drosophila* ovaries comprises of 16–20 ovarioles, which represent chains of progressively more and more mature egg chambers. At the anterior end of each ovariole lies the germarium, harboring two or three germline stem cells (GSCs), cushioned by somatic cap and terminal filament cells, which form the niche. Upon asymmetric division, the GSC self-renews and produces a daughter cell called the cystoblast, which divides four times synchronously to form a 16-cell interconnected germline cyst. Following enclosure by somatic follicle cells, the cyst embarks on a maturation program, which ultimately culminates in the production of an egg ready for fertilization (Spradling et al., 2011).

Current evidence indicates that the GSC state is maintained primarily by repression of differentiation-inducing pathways through extrinsic as well as GSC-intrinsic mechanisms (Slaidina and Lehmann, 2014; Spradling et al., 2011; Xie, 2013). Niche-derived Decapentaplegic (Dpp) and Glass-bottom boat (Gbb) activate bone morphogenetic protein (BMP) signaling in the GSCs leading to the transcriptional repression of *bag of marbles* (*bam*), a key differentiation-inducing factor (Chen and McKearin, 2003; McKearin and Spradling, 1990; Song et al., 2004; Xie and Spradling, 1998). Dynamic adherens junctions tether GSCs to the niche and relative E-cadherin levels at these contact points regulate the stemness potential of individual GSCs (Jin et al., 2008; Song et al., 2002). Within the GSCs, the translational repressor Nanos (nos) functions together with Pumilio (Pum) to prevent precocious GSC differentiation by repressing Bam-independent differentiation pathways, similar to its role in maintenance of primordial germ cells (PGCs) in the larvae (Forbes and Lehmann, 1998; Gilboa and Lehmann, 2004; Lin and Spradling, 1997; Szakmary et al., 2005; Wang and Lin, 2004).

Spatial constraints after GSC division cause posterior displacement of one of the daughter cells which, due to limited Dpp diffusion, upregulates *bam* transcription and starts the differentiation program. In the intervening period in which the GSC daughter has originated but Bam has not yet accumulated to critical levels, the cell is assumed to exist as a pre-cystoblast (Gilboa et al., 2003; Ohlstein and McKearin, 1997). Upon attaining Bam criticality, the pre-cystoblast, now a cystoblast, suppresses stemness-maintaining factors and commences the differentiation program through yet unknown mechanisms (Li et al., 2009a). Bam expression is necessary as well as sufficient to initiate this program, as *bam* mutant cells arrest at the pre-cystoblast stage and ectopic Bam expression forces premature GSC differentiation (McKearin and Ohlstein, 1995; Ohlstein and McKearin, 1997). Furthermore, even the larval PGCs develop cysts when exposed to Bam without ever becoming GSCs (Gilboa and Lehmann, 2004).

Forward and reverse genetics approaches have helped in uncovering these and several other molecular factors important for GSC maintenance and differentiation. Initial insights came from the analysis of effects of female sterile mutations on oogenesis (Perrimon et al., 1986; Schupbach and Wieschaus, 1991). Bam was identified in a P-element-based insertional mutagenesis screen as a sterility-inducing recessive mutation (Cooley et al., 1988; McKearin and Spradling, 1990). Lately, genome-wide RNAi screens have led to the identification of generic cellular processes such as ribosome biogenesis, protein synthesis and epigenetic regulation as important for the GSC state (Sanchez et al., 2016; Yan et al., 2014).

Although Bam is a vital GSC differentiation factor, it does not possess any known conserved protein domains that could allude to its mode of action. Microarray-based and RNA-seq transcriptomics studies of *bam* mutant ovaries have documented ensuing gene expression changes, which could be direct or indirect consequences of Bam inactivity (Kai et al., 2005; Gan et al., 2010). Several lines of evidence, however, indicate that Bam might act at the RNA-level in cohort with known RNA-binding proteins, if not alone, in promoting early germ cell maturation. For instance, it forms complexes with Benign gonial cell neoplasm (Bgcn), Mei-P26 and Sex-Lethal (Sxl) to effectuate repression of GSC-maintenance factors such as *nos* (Li et al., 2009a, 2013; Shen et al., 2009; Chau et al., 2012). Since Bgcn, Sxl and mei-P26 are themselves expressed at moderate-to-high levels within the GSCs, one could surmise that transient Bam expression in post-GSC cells serves to bring together these protein complexes for repressing specific transcripts which are required cell-autonomously within the GSCs for their maintenance. Consequently, Bam has been proposed to act through a translational ‘gating mechanism’ wherein its transient activity might confer mRNA target specificity (Slaidina and Lehmann, 2014).

In this study, we explore the function of Bam by transcriptionally profiling *bam* mutant ovaries using next generation sequencing (NGS) and further use GFP-tagged lines to find Bam-associated transcripts. Using these data, we define Bam-responsive networks important for GSC maintenance and differentiation. By comparative analysis, we further describe parallels between PGCs and GSCs and how Bam induction might cause these cells to differentiate. Our expression and RNA-binding data provide a resource for designing and conducting further studies on stem cell self-renewal and maturation.

## RESULTS

### Mutation in *bam* causes a massive change in gene expression in the female germ line

With only two or three GSCs per ovariole, GSC gene expression profiling faces a challenge in that insufficient amounts of RNA might be available for sequencing. This problem can be attenuated by utilizing GSC-like cells which emerge as a consequence of *bam* mutation or Dpp overexpression (McKearin and Ohlstein, 1995; Xie and Spradling, 1998). The *bam^−/−^* mitotic GSC-like cell population comprises of GSCs and undifferentiated pre-cystoblasts as evidenced by the existence of single and dumbbell-shaped spectrosome-containing cells (Fig. S1) (Kai et al., 2005; Ohlstein and McKearin, 1997). These cells exhibit delayed cytokinesis, like GSCs and PGCs, and retain the potential to populate the gonad leading to the establishment of a functional germline (Niki and Mahowald, 2003). Furthermore, the pre-cystoblasts do not comprise a transit-amplifying population and might just exist to reoccupy a vacant GSC niche, in case one arises (Gilboa et al., 2003). Thus, these cells appear to be at least functionally equivalent to GSCs.

**Fig. 1.**
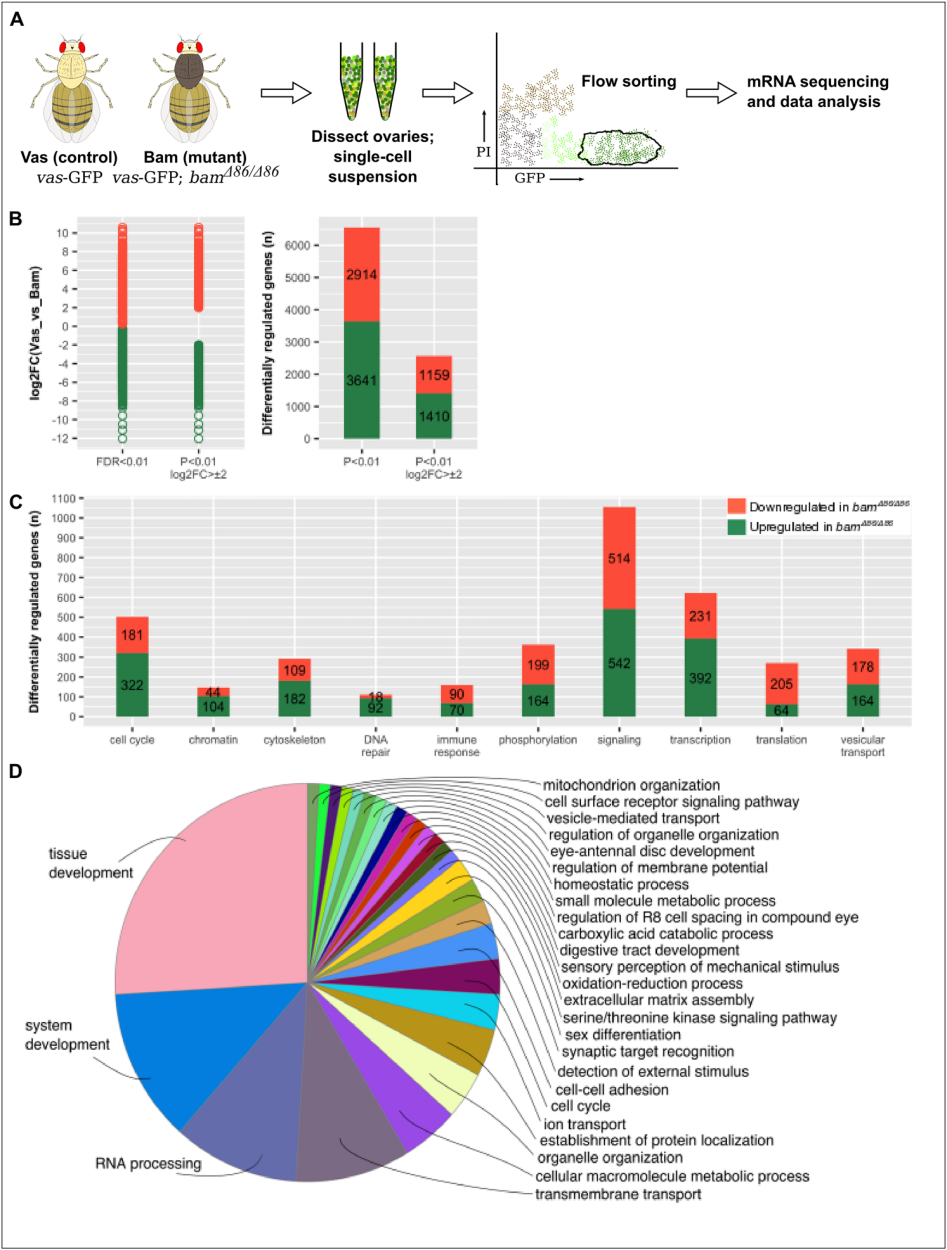
**Strategy and results summary for identifying the expression profile of GSC-like cells.** (A) FACS-based cell isolation and mRNA sequencing. (B) Distribution of the magnitude and frequencies of the differentially expressed genes at different filter [*P*-value and log_2_FC (fold change)] values. (C) Molecular function analysis of differentially regulated genes. (D) GO term analysis for biological processes of differentially regulated genes.

Since we were interested in identifying a Bam-responsive transcriptional program, we chose *vas*-GFP; *bam*^Δ*86*^/*bam*^Δ*86*^ flies (*bam^−/−^*) which have a near-complete deletion of the *bam* locus and in which the germline protein Vasa (Vas) is tagged with GFP. Further, to delineate the GSC-like transcriptome as accurately as possible, we used *vas*-GFP flies as our control population. In this way, we were able to compare the transcriptome of GSC-like cells to that of their differentiated progeny (Fig. 1A). Following FACS-sorting of PI^low^, GFP^high^ cells from *vas*-GFP; *bam*^Δ*86*^/*bam*^Δ*86*^ and *vas*-GFP flies, we isolated total RNA, enriched for mRNA, and subjected it to paired-end sequencing. Read alignment re-confirmed the *bam*^Δ*86*^ genotype (Fig. S2) and normalization indicated good sample clustering (Fig. S3).

In total, we found 6555 genes to be differentially regulated, which is approximately 43% of the *Drosophila* genome (Fig. 1B and Table S2). Of these, 3641 were upregulated while 2914 were downregulated in the *bam^−/−^* flies as compared to the control flies, with the magnitude of log_2_(fold change, FC) spanning from −9 to +11. A cursory analysis of molecular function of these genes indicated their involvement in almost all biological processes such as cell cycle regulation, chromatin remodeling, transcriptional and translational regulation, among others (Fig. 1C). Gene ontology (GO) term enrichment analysis for the biological processes in which these genes are involved showed major involvement in tissue development and RNA processing (Fig. 1D).

On expected lines in our data was the transcriptional upregulation of *nos* and *pum*, since their activity is cell-autonomously needed within the GSCs for averting differentiation – this also reaffirms the GSC-like character of our purified and profiled cell population. There was also an upregulation of transcripts encoding fusome components (*hu-li-tai-shao*, ɑ*-* and β*-spectrin*, and *ankyrin*) as would be required for maintaining this mitotic population (Lin et al., 1994). Also on anticipated lines was the upregulation of *Bgcn*, a DExH-box family of RNA-dependent helicases and a partner-in-crime required by Bam for repressing *nos* (Li et al., 2009a). Interestingly, we also observed upregulation of three transcripts specifically annotated for the GO term ‘male sex determination’ (GO:0030238) – *Phf7*, *chinmo* and *tra2,* which parallels previous reports of partial germline sex transformation in *sxl*, *ovarian tumor* (*otu*) and *bam* mutants (Staab et al., 1996; Wei et al., 1994; Shapiro-Kulnane et al., 2015).

### Several transcripts bind to Bam

To delve into the possible molecular functions of Bam, we surmised that a massive fate change transition such as GSC-to-commitment-to-differentiation would entail transcriptional as well as post-transcriptional regulation. In fact, Bam-dependent repression of *nos* in post-GSC cells is contingent upon sequences in the *nos* 3′-UTR (Li et al., 2009a). Along with Bgcn, Bam also binds to E-cadherin mRNA and represses its translation through its 3′-UTR in S2 cells (Shen et al., 2009). Bam-mediated translational repression has also been demonstrated during *Drosophila* spermatogenesis (Chen et al., 2014; Insco et al., 2012). More recently, it has been shown that the N-terminal region of Bam engages with the CAF40 subunit of the CCR4-NOT complex to effectuate target mRNA degradation (Sgromo et al., 2018).

**Fig. 2.**
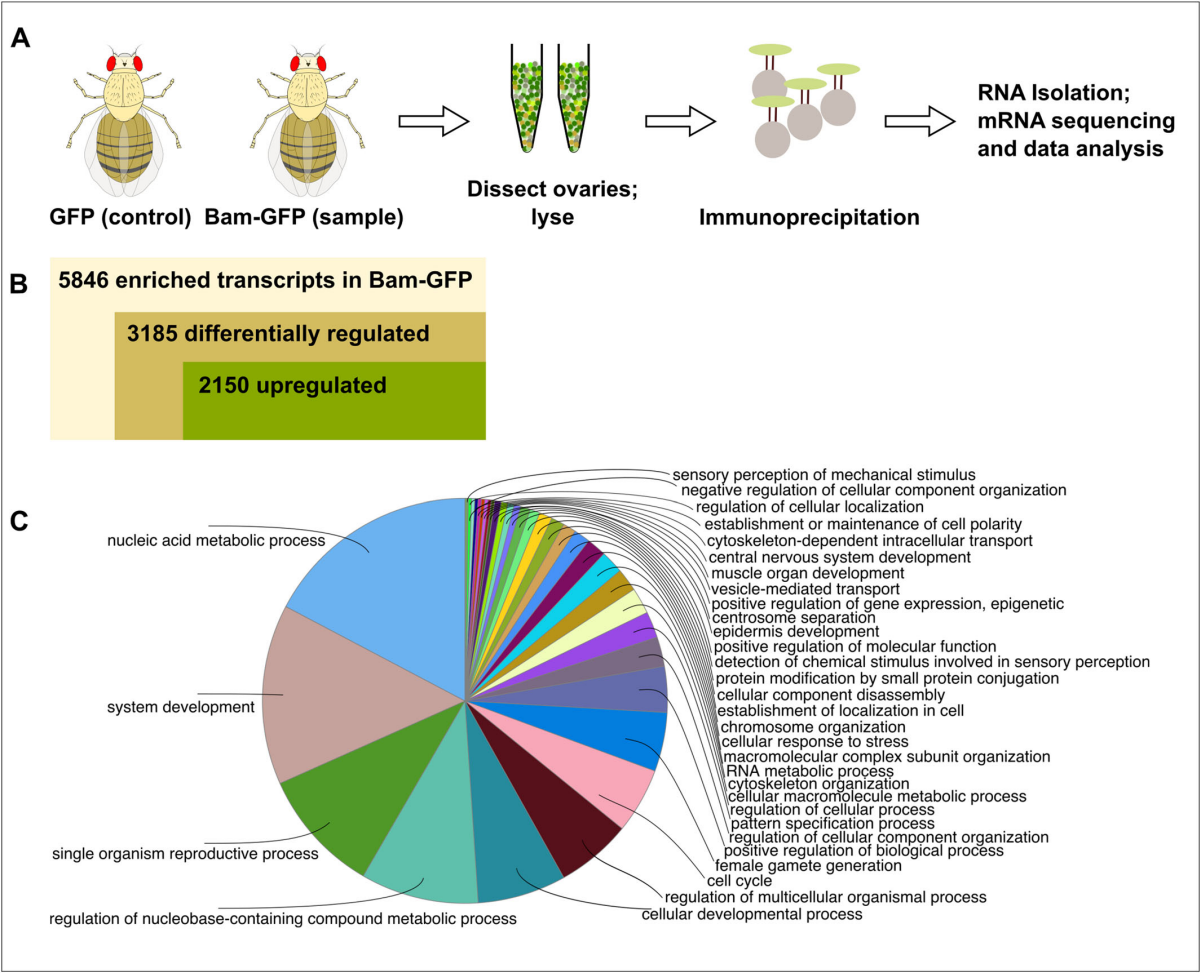
**Identification of transcripts bound to Bam-GFP.** (A) RIP-Seq strategy used to find Bam-bound transcripts. (B) Summary of RIP-seq results. (C) GO term analysis for biological processes of bound transcripts.

Since we noted differential regulations of a huge number of transcripts in our transcriptomics data, we hypothesized that transcriptionally upregulated Bam-bound transcripts would be attractive candidates as post-transcriptional Bam targets. For identifying these transcripts, we performed RNA-immunoprecipitation and sequencing (RIP-seq) by using the GFP-tagged Bam fTRG line and a *bam*-Gal4::*UASp*-GFP line as the control (Fig. 2A). We performed GFP-trap-bead-based immunoprecipitation followed by RNA isolation, mRNA sequencing and data analysis, and identified more than 5800 transcripts to be enriched in the Bam-GFP sample as compared to the control (Table S3). Of these, 1526 transcripts were significantly bound to Bam-GFP (*P*<0.05; Table S4). Interestingly, out of all the enriched transcripts, 3185 were also differentially regulated in our transcriptomics data (847, *P*<0.05) and of these, 2150 were upregulated (524, *P*<0.05; Tables S5 and S6). A GO term analysis for enriched biological processes on these bound and differentially regulated transcripts narrowed the scope of our previous GO term analysis (Figs 2C and 1D); apart from some generic processes such as nucleic acid metabolism and system development, we also saw enrichment for germline development processes such as reproduction, cellular development and gamete generation. Our RIP-seq strategy has thus aided us in refining the scope of our transcriptomics data.

Table 1 enlists a few of the mRNAs bound to Bam-GFP at a significance level of *P*<0.05. An interesting inclusion is that of the microRNA (miRNA) regulator and TRIM-NHL (tripartite motif and Ncl-1, HT2a and Lin-41 domain) family member Mei-P26, which is known to co-bind Bam and Bgcn in a tri-partite protein complex to repress *nos* mRNA in post-GSC cells (Li et al., 2013; Neumüller et al., 2008). Here, we found that *mei-P26* mRNA was bound to Bam-GFP. In transit-amplifying cells in *Drosophila* testes, Bam and Bgcn are known to repress *mei-P26* by binding to its 3′-UTR (Insco et al., 2012). These cells progress through an early Bam^ON^Mei-p26^ON^ state to a later Bam-mediated Bam^ON^Mei-P26^OFF^ state, indicating similar, but context-dependent, Bam-mediated translational regulation. Nevertheless, identification of this known Bam-bound mRNA underscores the potential functional relevance of our RIP-seq data.

**Table 1.**
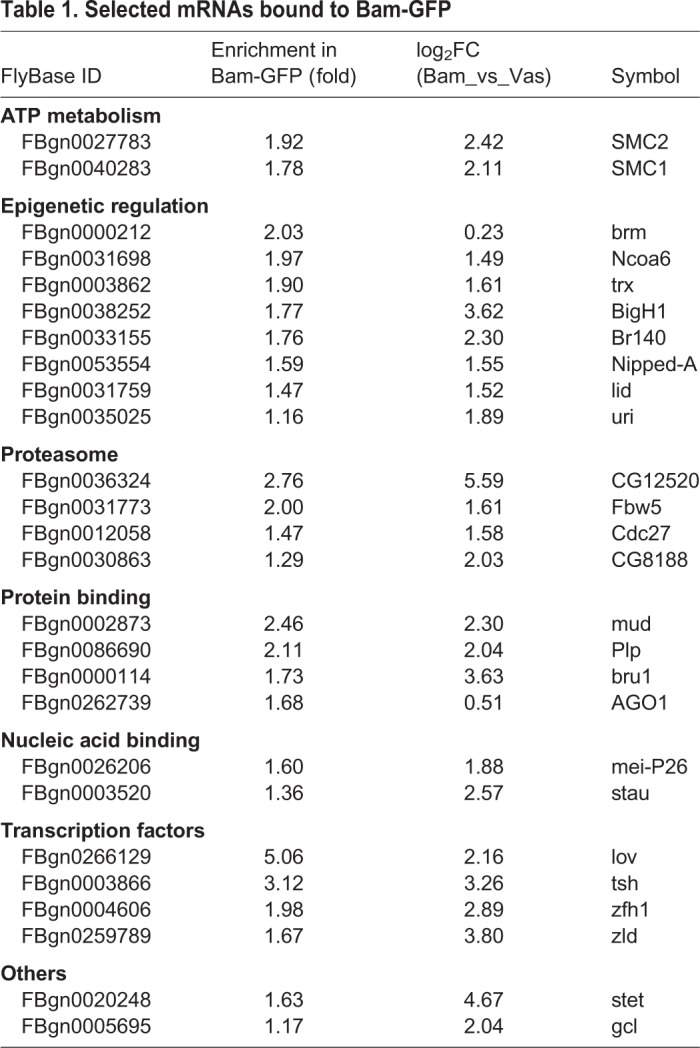
**Selected mRNAs bound to Bam-GFP**

### Bam-responsive networks required for GSC stemness and differentiation

By combining our transcriptomics and RIP-seq data, we next forecasted Bam-responsive protein networks important for GSC maintenance. For this, we assumed a positive correlation between gene expression and protein activity and utilized the protein complex enrichment analysis tool (COMPLEAT), which is an established framework for analyzing proteomics as well as high-throughput gene expression data. Using our Bam-bound and differentially expressed gene set as input, we identified 133 enriched complexes, of which 48 were downregulated and 85 were upregulated (Table S7). Next, we merged these networks using common protein nodes to generate undirected complex co-clusters and used a depiction for positive correspondence between node connectivity and node size to depict relative node connectivity. In these protein–protein interaction networks, a node represents a protein denoted by its symbol and the number of edges with which it is connected to determine its connectivity; the higher the connectivity, the larger the node size and vice versa.

**Fig. 3.**
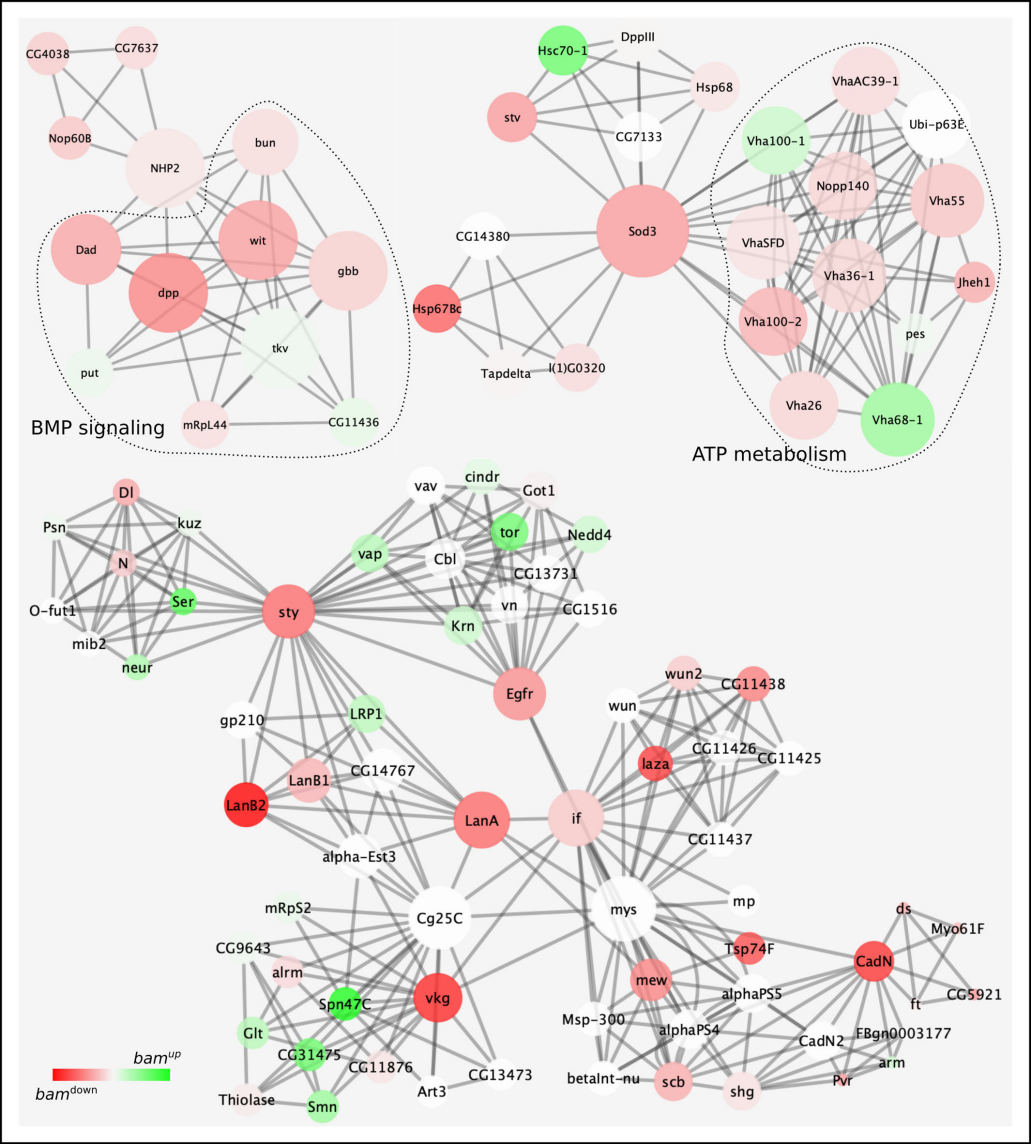
**Predicted protein networks downregulated in transcriptomics and RIP-seq data.** Protein–protein interaction networks are depicted using nodes and edges. Node connectivity directly corresponds to node size and node color gradient indicates up- (green) and downregulation (red) in *bam^−/−^*. For instance, BMP signaling and ATP metabolism complexes are interconnected with other complexes through protein nodes – NHP2 connects the BMP signaling complex to another tripartite complex involved in ribosomal biogenesis. Similarly, Superoxide dismutase 3 (Sod3) connects complexes involved in ATP metabolism and chaperone binding. Other major connecting nodes include sprouty (sty), Epidermal growth factor receptor (Egfr), Laminin A and B2 (Lan A and B2), inflated (if), Cadherin N (CadN) and Viking (vkg). White nodes are not differentially regulated in *bam^−/−^*.

Using this strategy, we were able to identify two facets which could be expected from a *bam^−/−^* germarium: (i) downregulation of a complex predicted to be involved in germline stem cell maintenance (FC3320, COMPLEAT classification scheme, Fig. S4). Driven by Rap1 GTPase, this complex effectuates EGFR signaling and has been shown to be required for anchoring *Drosophila* male GSCs to the niche, which is essential for preserving their stemness (Wang et al., 2006). (ii) Downregulation of BMP signaling (Fig. 3) – this is also expected since the Dpp signal is localized to about a one-cell diameter from the niche and profiling a complete germarium filled with GSC-like cells would show downregulation. Further, since Thickveins (Tkv, a type I BMP receptor) and Punt (Put, a type II BMP receptor) are expressed on GSCs and are individually upregulated in the data (Tables S2 and S7), this corroborates that the *bam*^Δ*86*^ population indeed consists of GSCs receptive to BMP signaling.

Since both of these facets act independently of Bam and are essential for GSC maintenance, one could surmise that other downregulated complexes might also be involved in the same process independently of Bam and appear downregulated as a consequence of accumulation of mitotic GSC-like cells (Fig. 3). Correspondingly, reactive oxygen species (ROS) levels are known regulators of stem cell fate across several stem cell types and tissues. For GSCs, Superoxide dismutase 3 (SOD3) appears to bridge protein folding and ATP metabolism along with maintaining ROS levels. Another interesting common node is Sprouty (sty) which connects complexes involved in organ morphogenesis, follicle cell development, EGFR signaling, and leads to integrin signaling which has a known role in ensuring male GSC-niche integrity and intestinal stem cell (ISC) homeostasis in *Drosophila* (Lin et al., 2013; Tanentzapf et al., 2007).

**Fig. 4.**
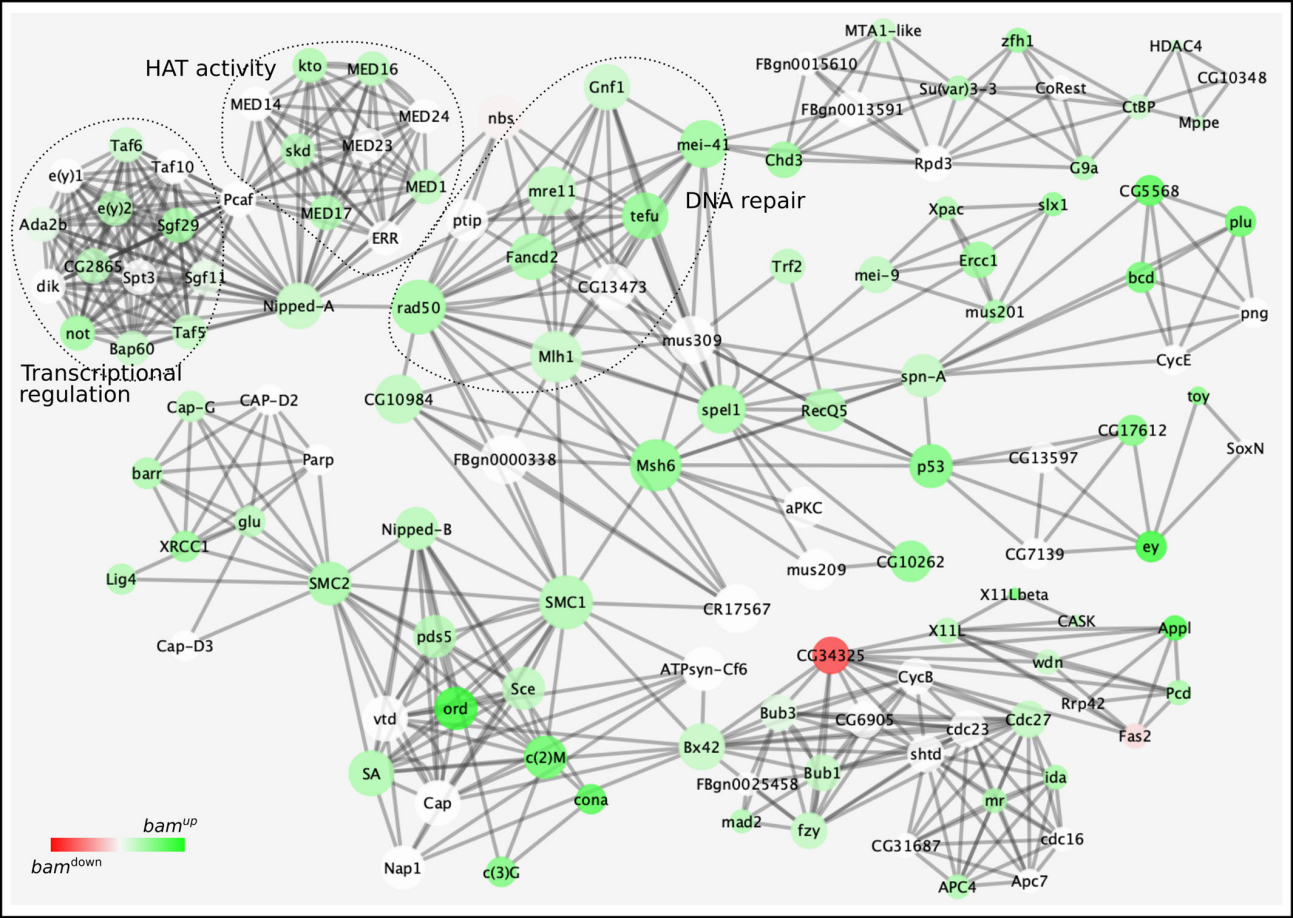
**Predicted protein networks upregulated in transcriptomics and RIP-seq data.** Protein–protein interaction networks are depicted using nodes and edges. Node connectivity directly corresponds to node size and node color gradient indicates up- (green) to downregulation (red) in *bam^−/−^*. Several highly connected protein nodes interconnect individual protein complexes: nipped-A bridges together complexes functioning in epigenetic regulation, transcription and DNA repair. Other highly connected nodes are: structural maintenance of chromosomes 1 and 2 (SMC 1 and 2), p53, Nipped-B, and Bx42. White nodes are not differentially regulated in *bam^−/−^*.

On the other end of the spectrum are the upregulated networks, which could potentially be responsive to Bam (Fig. 4). This would imply that their downregulation might or might not be essential for GSC maintenance, but it would be needed for initiating the Bam-induced differentiation program. A prominent node here appears to be Nipped-A, which connects complexes involved in transcriptional regulation, chromatin modification and DNA repair; fundamental changes in all of these processes are essential for a cell to embark on its maturation journey. Nipped-A is a subunit of the chromatin modifying histone acetyltransferase SAGA and Tip60 complexes in *Drosophila* and has been implicated in wing development and proliferation of ISCs (Gause et al., 2006; Tauc et al., 2017). Within the female germline, it has recently been shown to function as a part of the Tip60 complex for inducing expression of Bgcn, a known protein interactor of Bam (Li et al., 2009a; McCarthy et al., 2018). Here, our data also show Nipped-A to be a major focal point of several Bam-responsive complexes essential for differentiation.

### Comparative transcriptomics of *bam* mutant ovaries and PGCs

*Drosophila* embryonic and larval PGCs are maintained in a ‘naïve’ state through Bam-independent differentiation-repression mechanisms (Dansereau and Lasko, 2008). GSC formation begins in the late third-instar larval stage upon clonal expansion of PGCs juxtaposed with terminal filament and cap cells (Zhu and Xie, 2003). Since these PGCs possess the ability to form cysts as a consequence of ectopic Bam, they have been postulated to be functionally equivalent to GSCs in terms of maintenance of their stemness (Gilboa and Lehmann, 2004).

To arrive at this stemness-related molecular footprint of PGCs and GSCs, we compared the transcriptomes of *bam^−/−^* cells (our data) with zygotically transcribed transcripts in embryonic PGCs from a previously published microarray study (Siddiqui et al., 2012). Of the 720 transcripts significantly abundant in 3–7 h embryonic PGCs, 399 are also differentially regulated in *bam^−/−^* (Table S8). Furthermore, 287 of these are upregulated and GO term search shows enrichment for nitrogen compound metabolism indicating protein turnover and regulation (Fig. S5). Altogether, this analysis reveals potential candidates for common functional studies on PGCs and GSCs.

### Pilot scale functional RNAi screen reveals germline phenotypes

**Fig. 5.**
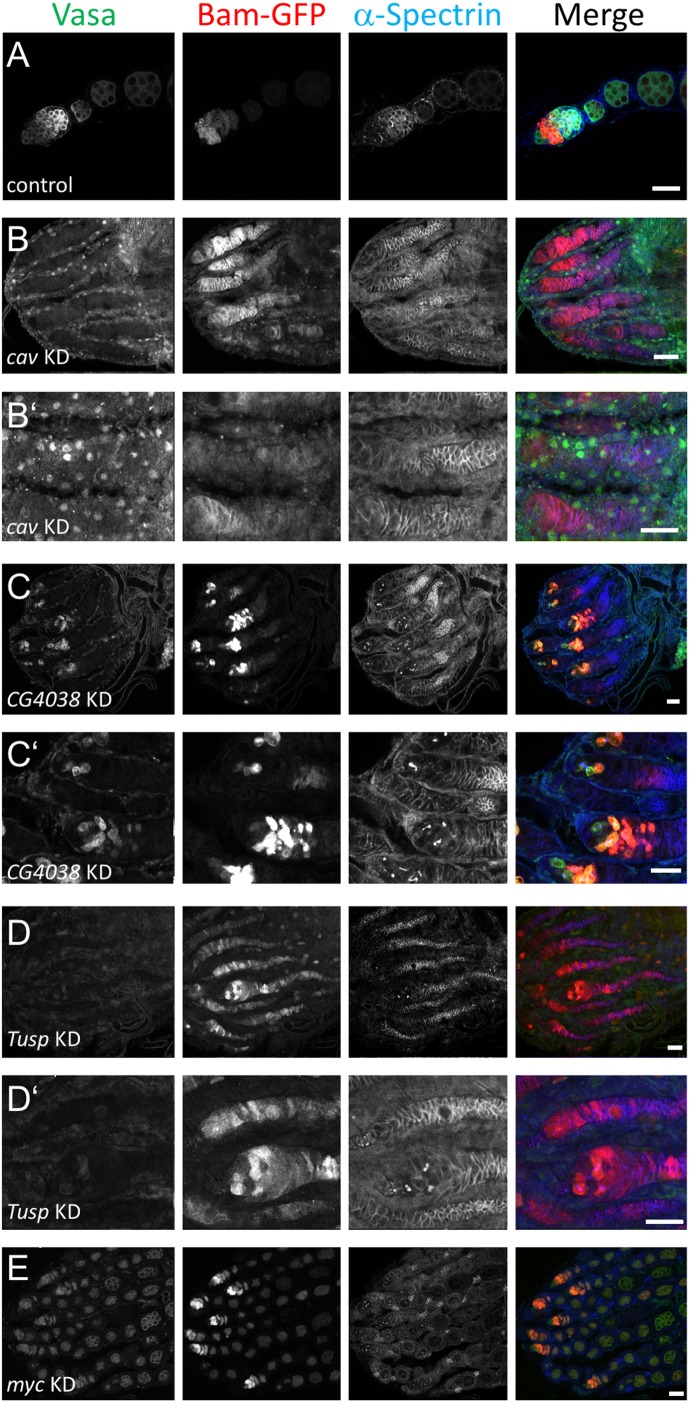
**Confocal micrographs showing phenotypes of selected genes after being knocked down using the *UAS*-Dcr2, *w^1118^*; *nosP*-GAL4-NGT40; *bamP*-GFP driver.** (A) Control flies are driver flies crossed with wild type (*w^−^*). The anterior part of a single ovariole with maturing egg-chambers stained for Vasa, GFP (Bam) and alpha-spectrin is shown. (B-E) RNAi knockdown phenotypes for cav (B,B′), *CG4038* (C,C′), *Tusp* (D,D′) and *myc* (E) stained as in A. Panels labeled with ' are higher magnification images of the respective genotypes. B′ is a digital magnification of B at a slightly different focal plane to illustrate non-specific anti-Vas staining within and around the apparently empty germaria. (B–E) The anterior tips of whole ovaries containing several ovarioles are shown. Anterior is to the left. Scale bars: 10 µm.

In order to gain functional insights from our transcriptomics data, we carried out a small-scale screen for some of the differentially regulated transcripts. We used virgin females from the germline driver *nos*-Gal4, together with a *bamP*-GFP transgene (*UAS*-Dcr2, w1118; *nosP*-GAL4-NGT40; *bamP*-GFP), to drive RNAi against specific genes. For microscopic visualization, we immunostained ovaries from the resulting progeny with anti-Vas, anti-GFP, and anti-ɑ-spectrin antibodies. As shown in Fig. 5, this approach resulted in the following phenotypes in a number of cases (Table S9; supplementary text for a summary of selected genes):

1. Empty germaria such as in *cav* (Fig. 5B,B′): no Vas^+^ or Bam^+^ cells indicating absence of germ cells and thus the germline; ɑ-spectrin^+^ empty follicles can also be observed.
2. Two-cell stage arrest as in *CG4038* and *Tusp* (Fig. 5C,C′,D,D′): where two-cell Vas^+^ and Bam^+^ clusters are connected by ɑ-spectrin^+^ spectrosomes. Absence of other stages indicates that these two-cell cysts were unable to mature further.
3. Oogenesis defects as in *myc* (Fig. 5E,E′): where germline development had proceeded to 8- or 16-cell cyst stages but further development was blocked.

.

One of the most comprehensive, RNAi-based functional genomics screens conducted earlier has also documented several of these phenotypes (Sanchez et al., 2016). Of the 779 germline-specific hits in this screen resulting in phenotypes of ‘no germline’, ‘germarium defects’, or ‘egg chamber defects’, 512 are also differentially regulated in our transcriptomics data (Table S10). Several of these are known protein-binding partners of Bam, such as Bgcn, and also Otu, which has been recently shown to cooperate with Bam for stabilizing Cyclin A – a fusome-associated protein (Ji et al., 2017; Lilly et al., 2000). Overall, this combined transcriptomic and functional analyses would serve to refine the scope of further experiments.

## DISCUSSION

*Drosophila* GSCs have been a long-standing model for studying stem cell division and differentiation. The only necessary and direct inducer of female GSC differentiation is Bam, which has been speculated to act post-transcriptionally. Here, we explore the transcriptional changes occurring as a consequence of absence of Bam using NGS and find almost a third of the known *Drosophila* genome to be differentially regulated. It is pertinent to point out here that we use the whole germline (*vas*-GFP^+^ cells) as a comparative population for calculating relative differential expression. Also, since we use a fluorescence-based sorting strategy, it is conceivable that cells expressing GFP at higher intensities – for instance, larger cells such as nurse cells or eight-cell cysts – would tend to be preferentially sorted. This would dilute the ‘ideal’ comparative scenario – comparing the transcriptome of a GSC with its direct descendent, the cystoblast – and might help explain this massive differential regulation. Furthermore, of particular note is the distinction between a Bam-induced transcriptional state, if it exists, versus a differentiation-induced transcriptional state. Given that GSCs can also undergo Bam-independent differentiation, our transcriptomics data are not able to distinguish between these two transcriptional states (Szakmary et al., 2005; Xi et al., 2005). It is thus plausible that the observed transcriptional differential expression might also be due to the ‘poised-to-differentiate’ state of the profiled cells, which are a mixed population of GSCs and pre-cystoblasts.

To distinguish between these two transcriptional states, we were able to further channel down our transcriptomics data using RIP-seq by identifying Bam-bound mRNAs, which may be subject to translational repression. However, in this experiment we had below average unique read mapping rates in the RIP-seq fastq data, ranging from 35% to 59% across our replicates (Table S1). This could either be due to fragmentation of immunoprecipitated RNA resulting in read mapping to multiple locations, or non-specific RNA binding, or molecular re-association after cell lysis. Insights from such a dataset could be limited as it is unlikely to capture ‘true’ read mapping leading to several insignificant results.

Furthermore, as can be seen in the RIP-seq data (Table S4), a lot of transcripts have very low (even zero) read counts in either of the replicates. However, in absence of a standard or universally-accepted read count filter to remove reads below a certain threshold value, we decided to include all the transcripts in which even one of the replicates exhibited read counts of greater than zero. Applying a random filter of, for instance, minimum 10 reads in each sample reduces this dataset by almost half, from 1526 to 822 significant results. Comparatively, Sxl-EGFP has been shown to be enriched with roughly 600 transcripts, albeit with a different enrichment calculation method (Ota et al., 2017). We thus leave it to the discretion of the larger scientific community to use our data with their considered opinion until a standardized paradigm for analyzing such a dataset is developed.

At the biological level, it is conceivable that an irreversible cell fate change from a stem cell to a differentiated cell would be driven from underlying transcriptional, and consequently translational, changes. Since all available evidence for the GSC-to-cystoblast cell fate change points towards post-transcriptional regulation and Bam is a major effector of this fate change, a large number of transcripts should be expected to associate with Bam and its binding partners. Of note here, however, is that because we used a Bam-GFP fusion protein in our immunoprecipitation strategy, and Bam's known mode of action is via binding to other RNA-binding proteins, this could change the Bam-complex composition in the wild-type state, leading to pseudo-binding.

Despite these aspects, we were able to identify known Bam mRNA binding partners and discover several new potential ones. Another significant point to be noted here is that due to the nature of RIP-seq itself, we cannot say anything about the repression activity of Bam from these data: an identified Bam-binder might or might not be repressed by it and this would call for further experimentation.

Several high-throughput functional genomics screens have utilized protein networks to capture the stem cell state across different *Drosophila* stem cell types, including GSCs (Neumüller et al., 2011; Sanchez et al., 2016; Yan et al., 2014; Zeng et al., 2015). Such protein networks illustrate the molecular machinery required for preserving stemness within the stem cell. We forecasted protein networks from our datasets and found several of them to be enriched in the *bam^−/−^* germaria. Importantly, however, our predicted protein networks lack any directionality, implying that cause–effect relationships cannot be deciphered. Also, since *bam^−/−^* germaria are far removed from a normally developing egg chamber, some of the networks could appear enriched due to perturbations resulting from heightened and aberrant mitotic activity, loss of contact with the niche, absence of wild-type feedback mechanisms between developing cysts, and atypical cellular stress levels.

Furthermore, node connectivity is just an indication of a protein being involved in multiple processes but does not give any information on how perturbations in its activity might affect GSC state. For instance, Nipped-A acts as a bridge between transcriptional regulation, HAT activity and DNA repair but how these processes function together is unclear. Simultaneous functional association studies will be necessary to elucidate the relative importance of nodal players with respect to the biological process involved in determining GSC state. On the whole, these networks capture the molecular state required to sustain *bam^−/−^* GSC-like cells and can serve as a foundation for conducting further studies.

The functional significance of our transcriptome data was demonstrated by the fact that many of the differentially regulated genes tested in our small-scale pilot screen affected germ line development upon knockdown by RNAi. Gene knockdown can result in empty germaria under two circumstances: if the gene is required for general cell survival, or if its activity is specifically needed within the GSCs for their maintenance. To distinguish between these scenarios, knockdown with Bam-Gal4 could be used, which is not active in GSCs but at all later stages of germ line cyst development. If the gene of interest was generally cell-lethal, knocking it down using *Bam*-Gal4 would allow survival of GSCs, but all other developmental stages in the germline would be absent. If the gene of interest was specifically required in GSCs, then knockdown using Bam-Gal4 should have no effect on germ line development. In this latter scenario, one can then probe if the gene is also acting along with, or independently of, Dpp signaling to repress Bam transcription within the GSCs.

Defects in GSC differentiation upon gene knockdown such as arrest at two- or four-cell stages can result if the gene is required specifically at these stages. If upregulated in the *bam^−/−^* transcriptome and enriched as a Bam-GFP binding partner, such a gene is a good candidate to be regulated post-transcriptionally through Bam. For all the tested genes exhibiting differentiation defects, we found them to bind to Bam-GFP in our RIP-seq dataset, although only *bruno* (*bru*) was significantly enriched at *P*<0.05. Bru is a translational repressor whose activity is needed from 4-cell cyst stage onwards to first repress *sxl*, and later *oskar*, through binding to Bruno response elements in their 3-UTRs (Filardo and Ephrussi, 2003; Parisi et al., 2001; Wang and Lin, 2007). Since Sxl and Bam expression is relatively high in cystoblasts and two-cell stages, it is plausible that they repress *bru* translation through an already established protein complex.

Altogether, our data provide a useful resource to identify biological processes differentially regulated between germ line stem cells and their differentiating progeny. However, the complexity of these differences is apparently higher than we initially expected. More sophisticated methods including single-cell transcriptomics will be required to shed more light on the important question of what makes stem cells so special.

## MATERIALS AND METHODS

### Fly stocks and genetics

All wild-type and transgenic flies were raised on standard *Drosophila* food at 25°C. The following flies were used: *vas*-GFP; *bam*^Δ*86*^/TM3,Sb (McKearin and Ohlstein, 1995) was a kind gift from Allan Spradling (Carnegie Institute of Washington, USA), *UAS*-Dcr2, *w^1118^*; *nosP*-GAL4-NGT40; *bamP*-GFP was kindly provided by Ruth Lehmann (New York University, USA), *bam*-Gal4 was generously gifted by Helen White-Cooper (Cardiff University, UK), *vas*-GFP (109171, Kyoto Stock Center), *bam*-fTRG (318001, Vienna *Drosophila* Research Center), and *UASp*-GFP. RNAi lines (details in Table S9) were obtained from the Vienna *Drosophila* Research Center and TRiP lines through the Bloomington *Drosophila* Stock Center (Dietzl et al., 2007; Ni et al., 2011).

### Single-cell suspension from ovaries and cell sorting

80–100 ovary pairs per genotype were hand-dissected in Schneider's media with 5% fetal calf serum on ice. The dissected ovaries were rinsed three times in chilled PBS and then incubated for 10 min at room temperature in a dissociation solution comprising of 2 mg/ml Collagenase in 0.5% Trypsin solution in PBS with intermittent vigorous shaking. After settlement of debris, the suspension was filtered through a 40 µm Filcon^®^ filter (BD Biosciences) followed by centrifugation at 500×***g*** for 5 min at 4°C. The resulting pellet was suspended in 1 ml of serum-free Schneider's medium containing 10 µg/ml propidium iodide (PI). After 30 min incubation at room temperature, samples were sorted on a BD FACSARIA™ II cell sorter using GFP^high^ and PI^low^ fluorescence intensity channels. Sample gating and acquisition were performed using BD FACSDIVA™ software (version 6.1.2).

### RNA isolation

RNA was isolated from sorted cells using the TRIzol™ (Thermo Fisher Scientific) purification method (Rio et al., 2010).

### mRNA sequencing

Paired-end RNA sequencing was performed at the Transcriptome and Genome Analysis Laboratory, University of Göttingen. mRNA was purified from 1 µg total RNA from each of the three replicates per genotype via poly-adenylated RNA selection using oligomer beads. Sequencing libraries were prepared using the Illumina TruSeq RNA kit. The resultant unstranded paired-end 100-bp mRNA libraries were multiplexed and run on Illumina HiSeq 2000. Sequencing data are accessible through NCBI GEO accession number GSE138987.

### Transcriptomic analysis

Quality control on the resulting reads was performed with FastQC (v0.11.7; Andrews, 2014). Reads were mapped to the *Drosophila* genome (FlyBase 6.05) with STAR (v2.4.0; Dobin et al., 2013). The resulting SAM files were converted to BAM files with SAMtools (v0.1.19; Li et al., 2009b) and viewed with the integrative genomics viewer (Robinson et al., 2011). Sorted BAM files were then used to call counts using Htseq (v0.11.0; Anders et al., 2015). Further processing of counts was done using Bioconductor (v3.7; Gentleman et al., 2004) in R (v3.5; R Core Team, 2014). Counts were normalized and differential expression was calculated using DESeq2 (v1.14.0; Love et al., 2014).

### GO term analysis

GO term analysis was carried out using the ClueGO plugin (v2.5.4; Bindea et al., 2009) in Cytoscape (v3.6; Shannon et al., 2003).

### RIP-seq

A protocol modified from (Loedige et al., 2015) and adapted to ovaries was used. Twenty-five pairs of dissected ovaries per genotype were lysed in a NET lysis buffer containing 50 mM Tris (pH 7.5), 150 mM NaCl, 5 mM EDTA, 0.5% NP-40, 10% Glycerol, 0.5 mM DTT and 2 µl of protease inhibitor mix using hand homogenization followed by incubation on ice for 20 min. The lysate was then cleared by centrifugation for 20 min at 15,000×***g*** at 4°C. Cleared lysate was incubated with 25 µl of ChromoTek GFP-Trap^®^ beads for 2.5 h on a rotating wheel at 4°C. Lysate-bound beads were washed three times with NET buffer containing 450 mM NaCl, followed by once with NET buffer containing 600 mM NaCl, and finally again with NET buffer containing 450 mM NaCl. RNA was then isolated using the TRIzol™ purification method and its quality was assessed on a Bioanalyzer. Following mRNA enrichment and library preparation, unpaired, single-end mRNA sequencing was carried out at the Cologne Center for Genomics, University of Cologne on an Illumina HiSeq 2000 machine. Sequencing data are accessible through NCBI GEO accession number GSE138987.

### RIP-seq data analysis

The raw fastq files were processed as for transcriptomic analysis until generation of counts for individual samples. For calculating transcript enrichment with respect to control, count data were fitted using an inverse beta binomial distribution for paired-sample testing in R (Pham and Jimenez, 2012).

### Network generation and analysis

Predicted and experimentally determined protein complexes were generated from RIP-seq binding data using COMPLEAT (Vinayagam et al., 2013). These complexes were merged based on common nodes (representing proteins) using the Network merge tool of Cytoscape and further analyzed using the Analyze network tool. The resulting merged complexes were superimposed using expression data from transcriptomic analysis.

### Immunofluorescence

Adult ovaries were dissected in cold PBS and then fixed in 4% formaldehyde in PBS for 10 min. Fixed ovaries were washed four times in PBT (PBS+0.2% Triton X-100) for 15 min per wash and then blocked with PBTB (PBT+0.2% Bovine serum albumine+5% Normal horse serum+0.05% Sodium azide) for 1 h at room temperature. Blocked samples were incubated with primary antibodies at respective dilutions in PBTB overnight at 4°C. Next day, ovaries were again washed four times in PBT for 15 min per wash followed by incubation with secondary antibodies at respective dilutions in PBTB for 2.5 h at room temperature. Samples were then washed four times in PBT for 15 min per wash with DAPI added in the penultimate wash. Finally, ovaries were hand dissected under a microscope and mounted on glass slides in NPGG (N-propyl Gallate+70% Glycerol). The following primary antibodies were used: mouse anti-ɑ-spectrin 1:10 (Developmental Studies Hybridoma Bank, DSHB, 3A9), rat anti-vasa 1:10 (DSHB), and rabbit anti-GFP 1:1000 (Invitrogen, A-11122). Alexa Fluor 488-, Alexa Fluor 567-, and Alexa Fluor 647-conjugated secondary antibodies (Invitrogen) were used at a dilution of 1:500. Samples were imaged on an LSM880 Airyscan Confocal microscope (Carl Zeiss Jena GmbH) and images were processed and assembled using Fiji and Inkscape (Rueden et al., 2017; Schindelin et al., 2012).

## Supplementary Material

Supplementary information

## References

[BIO046789C1] Anders S., Pyl P. T. and Huber W. (2015). HTSeq—a Python framework to work with high-throughput sequencing data. *Bioinformatics* 31, 166-169. 10.1093/bioinformatics/btu638 25260700 PMC4287950

[BIO046789C2] Andrews S. (2014). FastQC A Quality Control tool for High Throughput Sequence Data. Online available at: https://www.bioinformatics.babraham.ac.uk/projects/fastqc/.

[BIO046789C3] Baylis F. and McLeod C. (2007). The stem cell debate continues: the buying and selling of eggs for research. *J. Med. Ethics* 33, 726-731. 10.1136/jme.2007.022129 18055905 PMC2598212

[BIO046789C4] Bindea G., Mlecnik B., Hackl H., Charoentong P., Tosolini M., Kirilovsky A., Fridman W.-H., Pagès F., Trajanoski Z. and Galon J. (2009). ClueGO: a Cytoscape plug-in to decipher functionally grouped gene ontology and pathway annotation networks. *Bioinformatics* 25, 1091-1093. 10.1093/bioinformatics/btp101 19237447 PMC2666812

[BIO046789C80] Chau J., Shapiro-Kulnane L. and Salz H. K. (2012). Sex-lethal enables germline stem cell differentiation by downregulating Nanos protein levels during Drosophila oogenesis. *Proc. Natl. Acad. Sci. U. S. A.* 109, 9465-9470. 10.1073/pnas.1120473109 22645327 PMC3386055

[BIO046789C5] Chen D. and McKearin D. M. (2003). A discrete transcriptional silencer in the bam gene determines asymmetric division of the Drosophila germline stem cell. *Development* 130, 1159-1170. 10.1242/dev.00325 12571107

[BIO046789C6] Chen D., Wu C., Zhao S., Geng Q., Gao Y., Li X., Zhang Y. and Wang Z. (2014). Three RNA binding proteins form a complex to promote differentiation of germline stem cell lineage in Drosophila. *PLoS Genet.* 10, e1004797 10.1371/journal.pgen.1004797 25412508 PMC4238977

[BIO046789C7] Cooley L., Kelley R. and Spradling A. (1988). Insertional mutagenesis of the Drosophila genome with single P elements. *Science* 239, 1121-1128. 10.1126/science.2830671 2830671

[BIO046789C8] Dansereau D. A. and Lasko P. (2008). The development of germline stem cells in Drosophila. *Methods Mol. Biol. Clifton NJ* 450, 3-26. 10.1007/978-1-60327-214-8_1 PMC2729445 18370048

[BIO046789C9] Dietzl G., Chen D., Schnorrer F., Su K.-C., Barinova Y., Fellner M., Gasser B., Kinsey K., Oppel S., Scheiblauer S. et al. (2007). A genome-wide transgenic RNAi library for conditional gene inactivation in Drosophila. *Nature* 448, 151-156. 10.1038/nature05954 17625558

[BIO046789C10] Dobin A., Davis C. A., Schlesinger F., Drenkow J., Zaleski C., Jha S., Batut P., Chaisson M. and Gingeras T. R. (2013). STAR: ultrafast universal RNA-seq aligner. *Bioinformatics* 29, 15-21. 10.1093/bioinformatics/bts635 23104886 PMC3530905

[BIO046789C11] Evans M. J. and Kaufman M. H. (1981). Establishment in culture of pluripotential cells from mouse embryos. *Nature* 292, 154 10.1038/292154a0 7242681

[BIO046789C12] Filardo P. and Ephrussi A. (2003). Bruno regulates gurken during Drosophila oogenesis. *Mech. Dev.* 120, 289-297. 10.1016/S0925-4773(02)00454-9 12591598

[BIO046789C13] Forbes A. and Lehmann R. (1998). Nanos and Pumilio have critical roles in the development and function of Drosophila germline stem cells. *Development* 125, 679-690. 9435288 10.1242/dev.125.4.679

[BIO046789C81] Gan Q., Chepelev I., Wei G., Tarayrah L., Cui K., Zhao K. and Chen X. (2010). Dynamic regulation of alternative splicing and chromatin structure in Drosophila gonads revealed by RNA-seq. *Cell Res.* 20, 763-783. 10.1038/cr.2010.64 PMC2919574 20440302

[BIO046789C14] Gause M., Eissenberg J. C., Macrae A. F., Dorsett M., Misulovin Z. and Dorsett D. (2006). Nipped-A, the Tra1/TRRAP subunit of the Drosophila SAGA and Tip60 complexes, has multiple roles in Notch signaling during wing development. *Mol. Cell. Biol.* 26, 2347-2359. 10.1128/MCB.26.6.2347-2359.2006 16508010 PMC1430305

[BIO046789C15] Gentleman R. C., Carey V. J., Bates D. M., Bolstad B., Dettling M., Dudoit S., Ellis B., Gautier L., Ge Y., Gentry J. et al. (2004). Bioconductor: open software development for computational biology and bioinformatics. *Genome Biol.* 5, R80 10.1186/gb-2004-5-10-r80 15461798 PMC545600

[BIO046789C16] Gilboa L. and Lehmann R. (2004). Repression of primordial germ cell differentiation parallels germ line stem cell maintenance. *Curr. Biol.* 14, 981-986. 10.1016/j.cub.2004.05.049 15182671

[BIO046789C17] Gilboa L., Forbes A., Tazuke S. I., Fuller M. T. and Lehmann R. (2003). Germ line stem cell differentiation in Drosophila requires gap junctions and proceeds via an intermediate state. *Development* 130, 6625-6634. 10.1242/dev.00853 14660550

[BIO046789C18] Hunter P. (2008). The paradox of model organisms. The use of model organisms in research will continue despite their shortcomings. *EMBO Rep.* 9, 717-720. 10.1038/embor.2008.142 18670440 PMC2515201

[BIO046789C19] Insco M. L., Bailey A. S., Kim J., Olivares G. H., Wapinski O. L., Tam C. H. and Fuller M. T. (2012). A self-limiting switch based on translational control regulates the transition from proliferation to differentiation in an adult stem cell lineage. *Cell Stem Cell* 11, 689-700. 10.1016/j.stem.2012.08.012 23122292 PMC3833810

[BIO046789C20] Ji S., Li C., Hu L., Liu K., Mei J., Luo Y., Tao Y., Xia Z., Sun Q. and Chen D. (2017). Bam-dependent deubiquitinase complex can disrupt germ-line stem cell maintenance by targeting cyclin A. *Proc. Natl. Acad. Sci. USA* 114, 6316-6321. 10.1073/pnas.1619188114 28484036 PMC5474830

[BIO046789C21] Jin Z., Kirilly D., Weng C., Kawase E., Song X., Smith S., Schwartz J. and Xie T. (2008). Differentiation-defective stem cells outcompete normal stem cells for niche occupancy in the Drosophila ovary. *Cell Stem Cell* 2, 39-49. 10.1016/j.stem.2007.10.021 18371420 PMC8387725

[BIO046789C22] Kai T., Williams D. and Spradling A. C. (2005). The expression profile of purified Drosophila germline stem cells. *Dev. Biol.* 283, 486-502. 10.1016/j.ydbio.2005.04.018 15927177

[BIO046789C23] Li Y., Minor N. T., Park J. K., McKearin D. M. and Maines J. Z. (2009a). Bam and Bgcn antagonize Nanos-dependent germ-line stem cell maintenance. *Proc. Natl. Acad. Sci. USA* 106, 9304-9309. 10.1073/pnas.0901452106 19470484 PMC2695086

[BIO046789C24] Li H., Handsaker B., Wysoker A., Fennell T., Ruan J., Homer N., Marth G., Abecasis G. and Durbin R. and 1000 Genome Project Data Processing Subgroup (2009b). The sequence alignment/map format and SAMtools. *Bioinformatics* 25, 2078-2079. 10.1093/bioinformatics/btp352 19505943 PMC2723002

[BIO046789C25] Li Y., Zhang Q., Carreira-Rosario A., Maines J. Z., McKearin D. M. and Buszczak M. (2013). Mei-p26 cooperates with Bam, Bgcn and Sxl to promote early germline development in the Drosophila ovary. *PLoS ONE* 8, e58301 10.1371/journal.pone.0058301 23526974 PMC3603962

[BIO046789C26] Lilly M. A., de Cuevas M. and Spradling A. C. (2000). Cyclin A associates with the fusome during germline cyst formation in the Drosophila ovary. *Dev. Biol.* 218, 53-63. 10.1006/dbio.1999.9570 10644410

[BIO046789C27] Lin H. and Spradling A. C. (1997). A novel group of pumilio mutations affects the asymmetric division of germline stem cells in the Drosophila ovary. *Development* 124, 2463-2476. 9199372 10.1242/dev.124.12.2463

[BIO046789C28] Lin H., Yue L. and Spradling A. C. (1994). The Drosophila fusome, a germline-specific organelle, contains membrane skeletal proteins and functions in cyst formation. *Development* 120, 947-956. 7600970 10.1242/dev.120.4.947

[BIO046789C29] Lin G., Zhang X., Ren J., Pang Z., Wang C., Xu N. and Xi R. (2013). Integrin signaling is required for maintenance and proliferation of intestinal stem cells in Drosophila. *Dev. Biol.* 377, 177-187. 10.1016/j.ydbio.2013.01.032 23410794

[BIO046789C30] Loedige I., Jakob L., Treiber T., Ray D., Stotz M., Treiber N., Hennig J., Cook K. B., Morris Q., Hughes T. R. et al. (2015). The crystal structure of the NHL domain in complex with RNA reveals the molecular basis of drosophila brain-tumor-mediated gene regulation. *Cell Rep.* 13, 1206-1220. 10.1016/j.celrep.2015.09.068 26527002

[BIO046789C31] Love M. I., Huber W. and Anders S. (2014). Moderated estimation of fold change and dispersion for RNA-seq data with DESeq2. *Genome Biol.* 15, 550 10.1186/s13059-014-0550-8 25516281 PMC4302049

[BIO046789C32] McCarthy A., Deiulio A., Martin E. T., Upadhyay M. and Rangan P. (2018). Tip60 complex promotes expression of a differentiation factor to regulate germline differentiation in female Drosophila. *Mol. Biol. Cell* 29, 2933-2945. 10.1091/mbc.E18-06-0385 30230973 PMC6329907

[BIO046789C33] McKearin D. and Ohlstein B. (1995). A role for the Drosophila bag-of-marbles protein in the differentiation of cystoblasts from germline stem cells. *Development* 121, 2937-2947. 7555720 10.1242/dev.121.9.2937

[BIO046789C34] McKearin D. M. and Spradling A. C. (1990). bag-of-marbles: a Drosophila gene required to initiate both male and female gametogenesis. *Genes Dev.* 4, 2242-2251. 10.1101/gad.4.12b.2242 2279698

[BIO046789C35] McLaren A. (2001). Ethical and social considerations of stem cell research. *Nature* 414, 129 10.1038/35102194 11689959

[BIO046789C36] Morrison S. J. and Spradling A. C. (2008). Stem cells and niches: mechanisms that promote stem cell maintenance throughout life. *Cell* 132, 598-611. 10.1016/j.cell.2008.01.038 18295578 PMC4505728

[BIO046789C37] Morrison S. J., Shah N. M. and Anderson D. J. (1997). Regulatory mechanisms in stem cell biology. *Cell* 88, 287-298. 10.1016/S0092-8674(00)81867-X 9039255

[BIO046789C38] Neumüller R. A., Betschinger J., Fischer A., Bushati N., Poernbacher I., Mechtler K., Cohen S. M. and Knoblich J. A. (2008). Mei-P26 regulates microRNAs and cell growth in the Drosophila ovarian stem cell lineage. *Nature* 454, 241-245. 10.1038/nature07014 18528333 PMC2988194

[BIO046789C39] Neumüller R. A., Richter C., Fischer A., Novatchkova M., Neumüller K. G. and Knoblich J. A. (2011). Genome-wide analysis of self-renewal in drosophila neural stem cells by transgenic RNAi. *Cell Stem Cell* 8, 580-593. 10.1016/j.stem.2011.02.022 21549331 PMC3093620

[BIO046789C40] Ni J.-Q., Zhou R., Czech B., Liu L.-P., Holderbaum L., Yang-Zhou D., Shim H.-S., Tao R., Handler D., Karpowicz P. et al. (2011). A genome-scale shRNA resource for transgenic RNAi in Drosophila. *Nat. Methods* 8, 405-407. 10.1038/nmeth.1592 21460824 PMC3489273

[BIO046789C41] Niki Y. and Mahowald A. P. (2003). Ovarian cystocytes can repopulate the embryonic germ line and produce functional gametes. *Proc. Natl. Acad. Sci. USA* 100, 14042-14045. 10.1073/pnas.2235591100 14610282 PMC283542

[BIO046789C42] Ohlstein B. and McKearin D. (1997). Ectopic expression of the Drosophila Bam protein eliminates oogenic germline stem cells. *Development* 124, 3651-3662. 9342057 10.1242/dev.124.18.3651

[BIO046789C43] Ota R., Morita S., Sato M., Shigenobu S., Hayashi M. and Kobayashi S. (2017). Transcripts immunoprecipitated with Sxl protein in primordial germ cells of Drosophila embryos. *Dev. Growth Differ.* 59, 713-723. 10.1111/dgd.12408 29124738

[BIO046789C44] Parisi M. J., Deng W., Wang Z. and Lin H. (2001). The arrest gene is required for germline cyst formation during Drosophila oogenesis. *Genesis* 29, 196-209. 10.1002/gene.1024 11309853

[BIO046789C45] Perrimon N., Mohler D., Engstrom L. and Mahowald A. P. (1986). X-Linked female-sterile loci in Drosophila melanogaster. *Genetics* 113, 695-712. 3089870 10.1093/genetics/113.3.695 PMC1202863

[BIO046789C46] Pham T. V. and Jimenez C. R. (2012). An accurate paired sample test for count data. *Bioinformatics* 28, i596-i602. 10.1093/bioinformatics/bts394 22962487 PMC3436821

[BIO046789C47] R Core Team (2014). R: The R Project for Statistical Computing. Online available at: https://www.r-project.org/.

[BIO046789C48] Rio D. C., Ares M., Hannon G. J. and Nilsen T. W. (2010). Purification of RNA using TRIzol (TRI reagent). *Cold Spring Harb. Protoc.* 2010, pdb.prot5439 10.1101/pdb.prot5439 20516177

[BIO046789C49] Robinson J. T., Thorvaldsdóttir H., Winckler W., Guttman M., Lander E. S., Getz G. and Mesirov J. P. (2011). Integrative genomics viewer. *Nat. Biotechnol.* 29, 24-26. 10.1038/nbt.1754 21221095 PMC3346182

[BIO046789C50] Rueden C. T., Schindelin J., Hiner M. C., DeZonia B. E., Walter A. E., Arena E. T. and Eliceiri K. W. (2017). ImageJ2: ImageJ for the next generation of scientific image data. *BMC Bioinformatics* 18, 529 10.1186/s12859-017-1934-z 29187165 PMC5708080

[BIO046789C51] Sanchez C. G., Teixeira F. K., Czech B., Preall J. B., Zamparini A. L., Seifert J. R. K., Malone C. D., Hannon G. J. and Lehmann R. (2016). Regulation of ribosome biogenesis and protein synthesis controls germline stem cell differentiation. *Cell Stem Cell* 18, 276-290. 10.1016/j.stem.2015.11.004 26669894 PMC4744108

[BIO046789C52] Schindelin J., Arganda-Carreras I., Frise E., Kaynig V., Longair M., Pietzsch T., Preibisch S., Rueden C., Saalfeld S., Schmid B. et al. (2012). Fiji: an open-source platform for biological-image analysis. *Nat. Methods* 9, 676-682. 10.1038/nmeth.2019 22743772 PMC3855844

[BIO046789C53] Schupbach T. and Wieschaus E. (1991). Female sterile mutations on the second chromosome of drosophila melanogaster. II. mutations blocking oogenesis or altering egg morphology*. Genetics* 129, 1119-1136. 1783295 10.1093/genetics/129.4.1119 PMC1204776

[BIO046789C54] Sgromo A., Raisch T., Backhaus C., Keskeny C., Alva V., Weichenrieder O. and Izaurralde E. (2018). Drosophila Bag-of-marbles directly interacts with the CAF40 subunit of the CCR4-NOT complex to elicit repression of mRNA targets. *RNA* 24, 381-395. 10.1261/rna.064584.117 29255063 PMC5824357

[BIO046789C55] Shannon P., Markiel A., Ozier O., Baliga N. S., Wang J. T., Ramage D., Amin N., Schwikowski B. and Ideker T. (2003). Cytoscape: a software environment for integrated models of biomolecular interaction networks. *Genome Res.* 13, 2498-2504. 10.1101/gr.1239303 14597658 PMC403769

[BIO046789C82] Shapiro-Kulnane L., Smolko A. E. and Salz H. K. (2015). Maintenance of Drosophila germline stem cell sexual identity in oogenesis and tumorigenesis. *Development* 142, 1073-1082. 10.1242/dev.116590 25758221 PMC4360176

[BIO046789C56] Shen R., Weng C., Yu J. and Xie T. (2009). eIF4A controls germline stem cell self-renewal by directly inhibiting BAM function in the Drosophila ovary. *Proc. Natl. Acad. Sci. USA* 106, 11623-11628. 10.1073/pnas.0903325106 19556547 PMC2710669

[BIO046789C57] Siddiqui N. U., Li X., Luo H., Karaiskakis A., Hou H., Kislinger T., Westwood J. T., Morris Q. and Lipshitz H. D. (2012). Genome-wide analysis of the maternal-to-zygotic transition in Drosophila primordial germ cells. *Genome Biol.* 13, R11 10.1186/gb-2012-13-2-r11 22348290 PMC3334568

[BIO046789C58] Singec I., Jandial R., Crain A., Nikkhah G. and Snyder E. Y. (2007). The leading edge of stem cell therapeutics. *Annu. Rev. Med.* 58, 313-328. 10.1146/annurev.med.58.070605.115252 17100553

[BIO046789C59] Slaidina M. and Lehmann R. (2014). Translational control in germline stem cell development. *J. Cell Biol.* 207, 13-21. 10.1083/jcb.201407102 25313405 PMC4195835

[BIO046789C60] Song X., Zhu C.-H., Doan C. and Xie T. (2002). Germline stem cells anchored by adherens junctions in the Drosophila ovary niches. *Science* 296, 1855-1857. 10.1126/science.1069871 12052957

[BIO046789C61] Song X., Wong M. D., Kawase E., Xi R., Ding B. C., McCarthy J. J. and Xie T. (2004). Bmp signals from niche cells directly repress transcription of a differentiation-promoting gene, bag of marbles, in germline stem cells in the Drosophila ovary. *Development* 131, 1353-1364. 10.1242/dev.01026 14973291

[BIO046789C62] Spradling A., Fuller M. T., Braun R. E. and Yoshida S. (2011). Germline stem cells. *Cold Spring Harb. Perspect. Biol.* 3, a002642 10.1101/cshperspect.a002642 21791699 PMC3220357

[BIO046789C63] Staab S., Heller A. and Steinmann-Zwicky M. (1996). Somatic sex-determining signals act on XX germ cells in Drosophila embryos. *Development* 122, 4065-4071. 9012526 10.1242/dev.122.12.4065

[BIO046789C64] Szakmary A., Cox D. N., Wang Z. and Lin H. (2005). Regulatory relationship among piwi, pumilio, and bag-of-marbles in Drosophila germline stem cell self-renewal and differentiation. *Curr. Biol.* 15, 171-178. 10.1016/j.cub.2005.01.005 15668175

[BIO046789C65] Tanentzapf G., Devenport D., Godt D. and Brown N. H. (2007). Integrin-dependent anchoring of a stem-cell niche. *Nat. Cell Biol.* 9, 1413-1418. 10.1038/ncb1660 17982446 PMC3529653

[BIO046789C66] Tauc H. M., Tasdogan A., Meyer P. and Pandur P. (2017). Nipped-A regulates intestinal stem cell proliferation in Drosophila. *Development* 144, 612-623. 10.1242/dev.142703 28196804

[BIO046789C67] Vinayagam A., Hu Y., Kulkarni M., Roesel C., Sopko R., Mohr S. E. and Perrimon N. (2013). Protein complex-based analysis framework for high-throughput data sets. *Sci. Signal.* 6, rs5 10.1126/scisignal.2003629 23443684 PMC3756668

[BIO046789C68] Wang Z. and Lin H. (2004). Nanos maintains germline stem cell self-renewal by preventing differentiation. *Science* 303, 2016-2019. 10.1126/science.1093983 14976263

[BIO046789C69] Wang Z. and Lin H. (2007). Sex-lethal is a target of Bruno-mediated translational repression in promoting the differentiation of stem cell progeny during Drosophila oogenesis. *Dev. Biol.* 302, 160-168. 10.1016/j.ydbio.2006.09.016 17067567 PMC1904479

[BIO046789C70] Wang H., Singh S. R., Zheng Z., Oh S.-W., Chen X., Edwards K. and Hou S. X. (2006). Rap-GEF signaling controls stem cell anchoring to their niche through regulating DE-cadherin-mediated cell adhesion in the Drosophila testis. *Dev. Cell* 10, 117-126. 10.1016/j.devcel.2005.11.004 16399083

[BIO046789C71] Watt F. M. and Driskell R. R. (2010). The therapeutic potential of stem cells. *Philos. Trans. R. Soc. B Biol. Sci.* 365, 155-163. 10.1098/rstb.2009.0149 PMC2842697 20008393

[BIO046789C72] Wei G., Oliver B., Pauli D. and Mahowald A. P. (1994). Evidence for sex transformation of germline cells in ovarian tumor mutants of Drosophila. *Dev. Biol.* 161, 318-320. 10.1006/dbio.1994.1032 8293883

[BIO046789C73] Xi R., Doan C., Liu D. and Xie T. (2005). Pelota controls self-renewal of germline stem cells by repressing a Bam-independent differentiation pathway. *Development* 132, 5365-5374. 10.1242/dev.02151 16280348

[BIO046789C74] Xie T. (2013). Control of germline stem cell self-renewal and differentiation in the Drosophila ovary: concerted actions of niche signals and intrinsic factors. *Wiley Interdiscip. Rev. Dev. Biol.* 2, 261-273. 10.1002/wdev.60 24009036

[BIO046789C75] Xie T. and Spradling A. C. (1998). decapentaplegic is essential for the maintenance and division of germline stem cells in the Drosophila ovary. *Cell* 94, 251-260. 10.1016/S0092-8674(00)81424-5 9695953

[BIO046789C76] Xie T. and Spradling A. C. (2000). A niche maintaining germ line stem cells in the Drosophila ovary. *Science* 290, 328-330. 10.1126/science.290.5490.328 11030649

[BIO046789C77] Yan D., Neumüller R. A., Buckner M., Ayers K., Li H., Hu Y., Yang-Zhou D., Pan L., Wang X., Kelley C. et al. (2014). A regulatory network of Drosophila germline stem cell self-renewal. *Dev. Cell* 28, 459-473. 10.1016/j.devcel.2014.01.020 24576427 PMC3998650

[BIO046789C78] Zeng X., Han L., Singh S. R., Liu H., Neumüller R. A., Yan D., Hu Y., Liu Y., Liu W., Lin X. et al. (2015). Genome-wide RNAi screen identifies networks involved in intestinal stem cell regulation in Drosophila. *Cell Rep.* 10, 1226-1238. 10.1016/j.celrep.2015.01.051 25704823 PMC4420031

[BIO046789C79] Zhu C.-H. and Xie T. (2003). Clonal expansion of ovarian germline stem cells during niche formation in Drosophila. *Development* 130, 2579-2588. 10.1242/dev.00499 12736203

