# Supplementary material for: Molecular profiling of stem cell-like female germ line cells in Drosophila delineates networks important for stemness and differentiation
Source: Biol Open. 2019 Nov 12;8(11):bio046789. doi: 10.1242/bio.046789 (PMC6899027; doi:10.1242/bio.046789)
Supplement: Supplementary information [file biolopen-8-046789-s1.pdf]

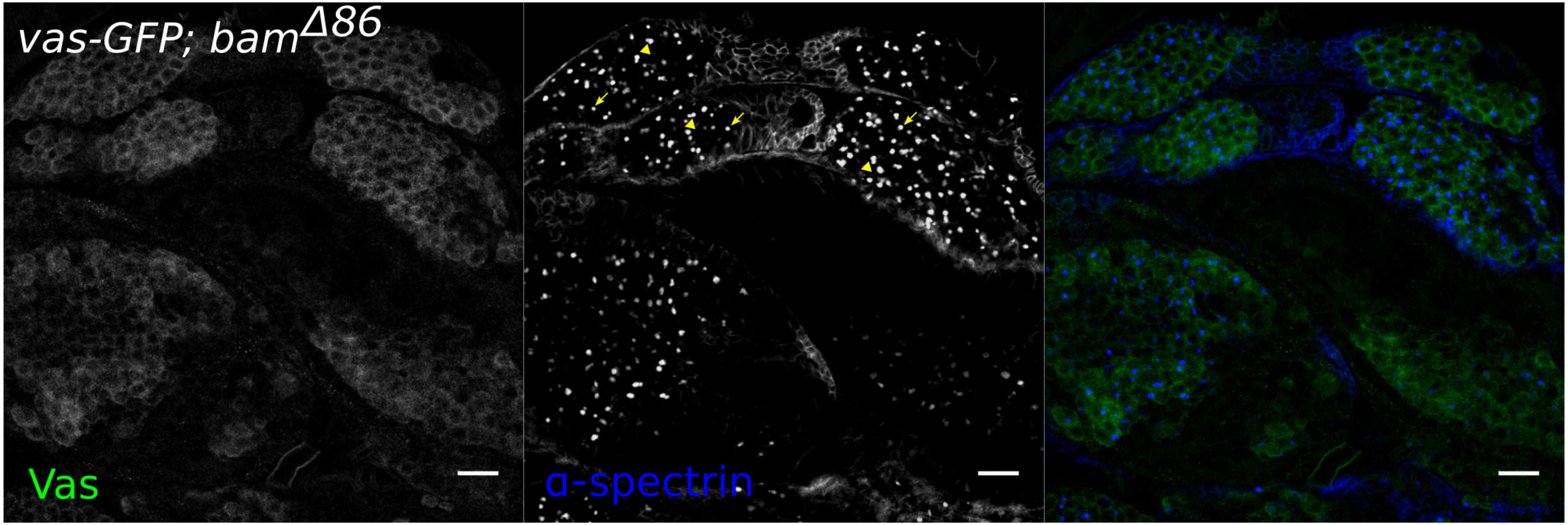

Figure S1. Confocal micrograph of *vas*-GFP; *bam*$^{\Delta 86}$/*bam*$^{\Delta 86}$ germaria stained with anti-GFP for assessing Vas protein distribution and anti-α-spectrin which marks the spectrosomes; single spectrosomes are indicated by yellow arrows and dumbbell-shaped spectrosomes by yellow triangles.

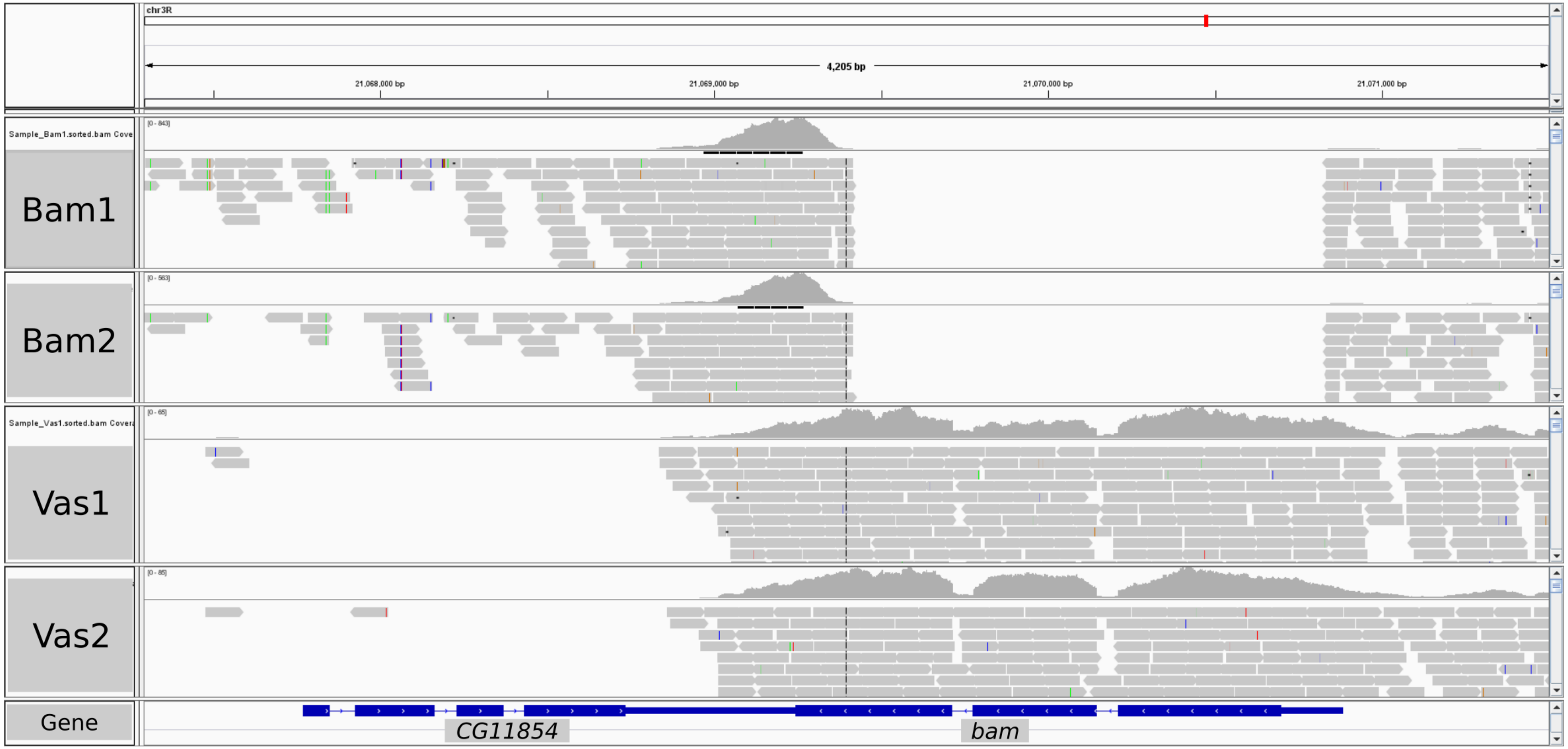

Figure S2. Integrative genomics viewer snapshot of reads aligning to the *bam* locus in *vas*-GFP; $bam^{\Delta86}/bam^{\Delta86}$ flies (Bam1 and Bam2) and vas-GFP flies (Vas1 and Vas2). Almost complete absence aligned reads at the *bam* locus indicates near-complete deletion in the $bam^{\Delta86}$ mutant.

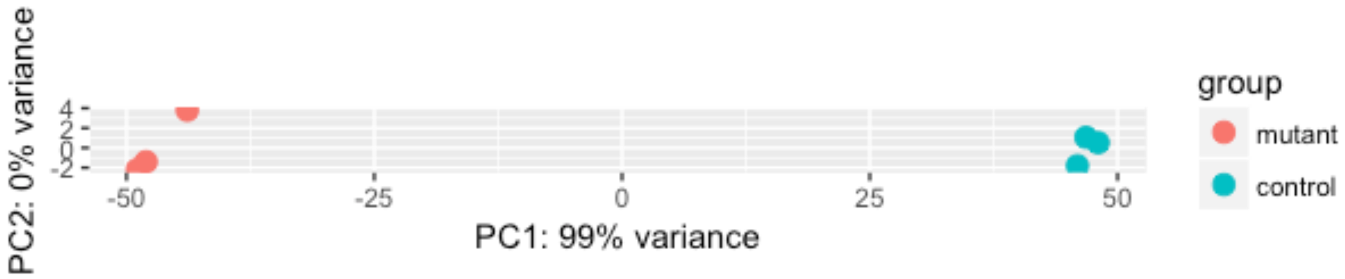

Figure S3. PCA plot of vas-GFP; $bam^{\Delta 86}$ and vas-GFP (control) showing efficient sample clustering.

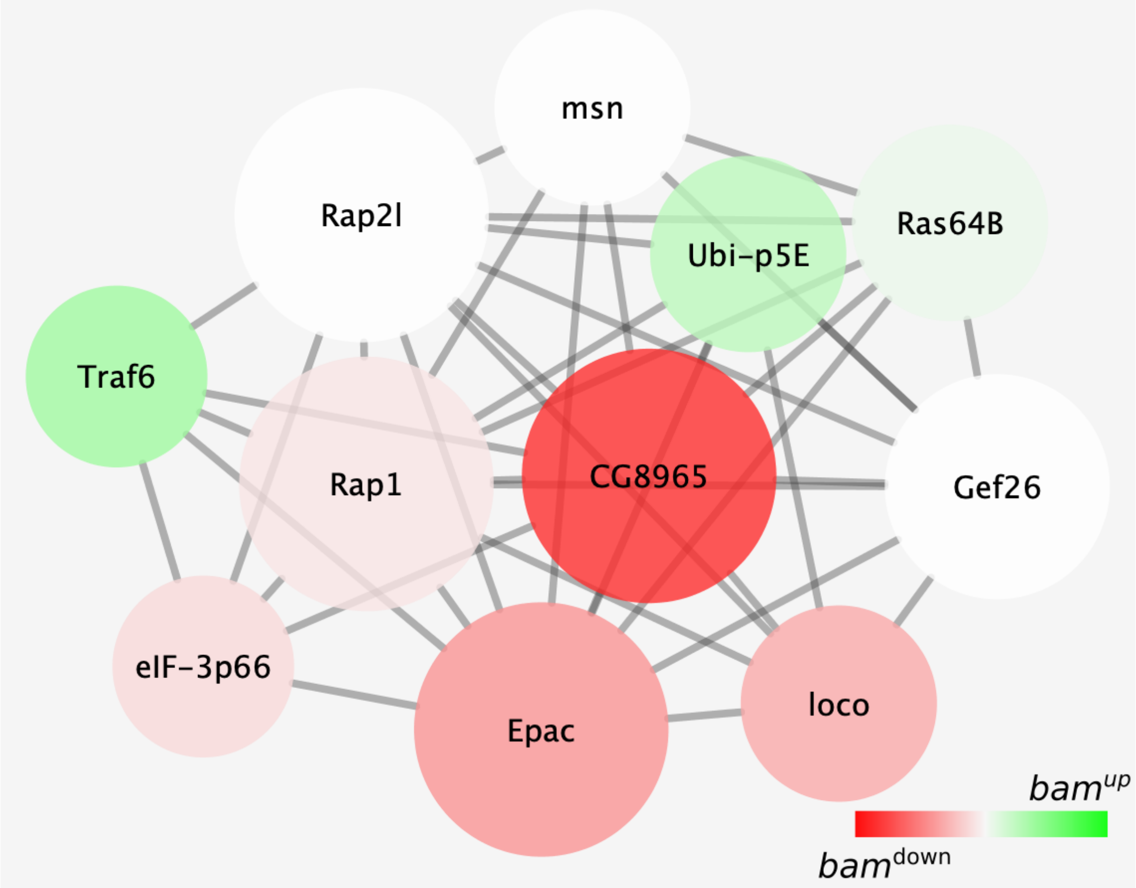

Figure S4. A predicted complex involved in GSC maintenance.

Biology Open • Supplementary information

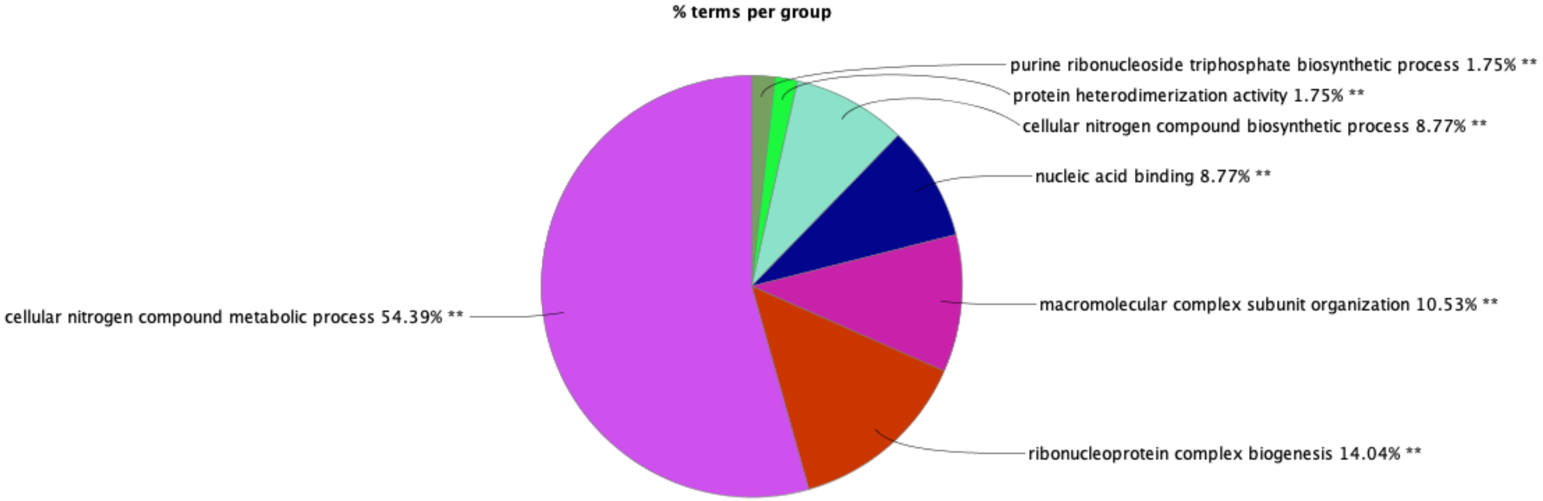

Figure S5. GO term analysis of common transcripts between *bam*$^{-/-}$ cells and PGCs.

Supplementary Text. Related to Figure 5.

Caravaggio (cav). *Cav* encodes the *Drosophila* HP1/ORC Associated Protein (HOAP) which is required for telomere capping. *Cav*$^{-/-}$ flies are larval/pupal lethal and display telomere fusions in larval brain cells (Cenci et al., 2003). The tripartite complex serves to regulate heterochromatin which, in turn, affects nuclear modelling and centrosomal function, and may contribute to species-specific differentiation mechanisms (Dernburg et al., 1996; Dobie et al., 1999).

CG4038. *CG4038* encodes a putative snoRNA binding protein involved in rRNA pseudouridine synthesis (FlyBase Gene Report: Dmel\CG4038).

Tusp. Tusp is a WD40-repeat containing protein required for SNARE complex formation (Yoon et al., 2017). It is a pan-neuronal protein and *tusp*$^{-/-}$ display temperature-sensitive paralysis.

Myc. *Drosophila* Myc is a proto-oncogene involved in cellular growth. Ovarian germline stem cells (GSCs) exhibit high levels of Myc and its expression reduces as they progress through the pre-cystoblast to the cystoblast stage. Among the GSCs, relatively high Myc levels appear to confer a preferential advantage for niche occupancy (Rhiner et al., 2009). Myc is also required cell-autonomously within the germline for cell growth and endoreplication (Maines et al., 2004).

Biology Open • Supplementary information

Table S1.

Uniquely mapped reads (%) resulting from mapping RIP-Seq experiment fastq files to the *Drosophila* genome. These read percentages are reported by the STAR aligner in its mapping reports (Log.final.out.txt files).

Sample	Uniquely mapped reads (%)
Bam-GFP1	46.8
Bam-GFP2	39.06
Bam-GFP3	59.01
GFP1	40.01
GFP2	34.96
GFP3	45.78

Table S2. List of genes differentially regulated in the vas-GFP; bamΔ86/bamΔ86 flies as compared to vas-GFP (control) flies. The results emanate from DESeq2 analysis of mapped and counted reads after paired-end mRNA sequencing. Log2FC(Bam-vs_Vas) indicates the log2(Fold Change) values of respective genes – a positive value indicates upregulation in vas-GFP; bamΔ86/bamΔ86 and a negative value indicates downregulation in vas-GFP; bamΔ86/ bamΔ86 as compared to vas-GFP (control). padj is the adjusted p-value reported by DESeq2 after Benjamini & Hochberg correction.

Click here to Download Table S2

Table S3. List of genes differentially enriched in Bam-GFP as compared to bam-Gal4::UASp-GFP (control) flies. These results arise from data processing of RIP-Seq experimental data using the inverse beta binomial test . Bam-GFP1-3 and GFP1-3 are biological replicates and the numbers represent read count data for that particular gene in that particular replicate. out$FC and out$p.value are the fold changes and p-values obtained after performing the test, repectively.

Click here to Download Table S3

Table S4. List of genes significantly differentially enriched in Bam-GFP as compared to bam-Gal4::UASp-GFP (control) flies at p-value < 0.05. Data originates from Table S3.

Click here to Download Table S4

Table S5. List of genes common between transcriptomics and RIP-Seq datasets. These results arise from intersection of data in the transcriptomics experiment (Table S2) and the RIP-Seq experiment (Table S3).

Click here to Download Table S5

Biology Open • Supplementary information

Table S6. List of genes common between transcriptomics and RIP-Seq datasets at p-value < 0.05. Data originates from Table S5.

Click here to Download Table S6

Table S7. List of protein complexes enriched at p-value < 0.05 using genes common between transcriptomics and RIP-Seq datasets as the input data. Complex ID is the protein complex identification tag reported by COMPLEAT; size is the relative protein complex size in terms of number of networked proteins; score (logFC) is computed by COMPLEAT based on the interquartile mean of individual complex members and is an indicator of relative complex enrichment.

Click here to Download Table S7

Table S8. List of transcripts common between our transcriptomics data and transcripts abundant in 3-7 h embryonic PGCs (Siddiqui et al., 2012). Data originates from our file Table S2 and updated FlyBase IDs from Additional File 19 of the (Siddiqui et al., 2012) data.

Click here to Download Table S8

Table S9. Phenotypic observations from a pilot RNAi screen conducted using *UAS*-Dcr2, w1118; *nosP*-GAL4-NGT40; *bamP*-GFP flies.

Click here to Download Table S9

Table S10. List of transcripts common between our transcriptomics data and positive hits in the functional genomic screen conducted by (Sanchez et al., 2016). Data originates from our file Table S2 and updated FlyBase IDs from Supplementary FileS1 of the (Sanchez et al., 2016) data.

Click here to Download Table S10

Biology Open • Supplementary information

Supplementary References

Cenci, G., Siriaco, G., Raffa, G. D., Kellum, R. and Gatti, M. (2003). The Drosophila HOAP protein is required for telomere capping. *Nature Cell Biology* **5**, 82.

Dernburg, A. F., Sedat, J. W. and Hawley, R. S. (1996). Direct Evidence of a Role for Heterochromatin in Meiotic Chromosome Segregation. *Cell* **86**, 135–146.

Dobie, K. W., Hari, K. L., Maggert, K. A. and Karpen, G. H. (1999). Centromere proteins and chromosome inheritance: a complex affair. *Current Opinion in Genetics & Development* **9**, 206–217.

FlyBase Gene Report: Dmel\CG4038.

Maines, J. Z., Stevens, L. M., Tong, X. and Stein, D. (2004). Drosophila dMyc is required for ovary cell growth and endoreplication. *Development* **131**, 775–786.

Rhiner, C., Díaz, B., Portela, M., Poyatos, J. F., Fernández-Ruiz, I., López-Gay, J. M., Gerlitz, O. and Moreno, E. (2009). Persistent competition among stem cells and their daughters in the Drosophila ovary germline niche. *Development* **136**, 995–1006.

Yoon, E. J., Jeong, Y. T., Lee, J. E., Moon, S. J. and Kim, C. H. (2017). Tubby domain superfamily protein is required for the formation of the 7S SNARE complex in Drosophila. *Biochemical and Biophysical Research Communications* **482**, 814–820.
